# The steroid hormone ecdysone regulates growth rate in response to oxygen availability

**DOI:** 10.1038/s41598-022-08563-9

**Published:** 2022-03-18

**Authors:** George P. Kapali, Viviane Callier, Samuel J. L. Gascoigne, Jon F. Harrison, Alexander W. Shingleton

**Affiliations:** 1 Department of Biological Sciences, University of Illinois Chicago, 840 West Taylor Street, Chicago, IL 60607 USA; 2 School of Life Sciences, Arizona State University, Tempe, AZ 85287-4501 USA; 3 Department of Biology, Lake Forest College, 555 North Sheridan Road, Lake Forest, IL 60045 USA

**Keywords:** Developmental biology, Respiration

## Abstract

In almost all animals, physiologically low oxygen (hypoxia) during development slows growth and reduces adult body size. The developmental mechanisms that determine growth under hypoxic conditions are, however, poorly understood. Here we show that the growth and body size response to moderate hypoxia (10% O_2_) in *Drosophila melanogaster* is systemically regulated via the steroid hormone ecdysone. Hypoxia increases level of circulating ecdysone and inhibition of ecdysone synthesis ameliorates the negative effect of low oxygen on growth. We also show that the effect of ecdysone on growth under hypoxia is through suppression of the insulin/IGF-signaling pathway, via increased expression of the insulin-binding protein *Imp-L2*. These data indicate that growth suppression in hypoxic *Drosophila* larvae is accomplished by a systemic endocrine mechanism that overlaps with the mechanism that slows growth at low nutrition. This suggests the existence of growth-regulatory mechanisms that respond to general environmental perturbation rather than individual environmental factors.

## Introduction

Low physiological oxygen, referred to as hypoxia, is known to reduce both growth rate and final body size in almost all animals^1–3^. Despite the near ubiquity of this phenomenon, however, we still have a poor understanding of how oxygen regulates organismal growth in multicellular animals. Decades of research has established that low oxygen reduces cellular respiration and slows ATP-dependent cellular processes, including cell proliferation. More recently, the discovery of oxygen-sensitive transcription factors, known as hypoxia inducible factors (HIFs), has further increased our understanding of the cellular response to low oxygen^4,5^. While the cell-autonomous response to hypoxia likely plays a role in regulating the effect of oxygen on body size, more recent research has implicated systemic mechanisms in this process also, using *Drosophila* as a model organism. Specifically, low oxygen appears to suppress growth and final body size in *Drosophila* via the insulin/insulin-growth factor signaling (IIS) pathway, through the actions of HIF-signaling in the fat body (the *Drosophila* equivalent of the liver/adipose tissue)^6^. The IIS pathway canonically controls growth in response to nutrition, and the observation that hypoxia also suppresses IIS activity suggests that the growth-response to hypoxia and low nutrition share a common regulatory mechanism^7–9^. The effect of nutrition on growth and final body size is not solely regulated by IIS, however, but involves other hormones, in particular the steroid ecdysone^1,10–12^. An open question, therefore, is whether ecdysone is also involved in regulating growth and final body size in response to hypoxia.

Ecdysone canonically regulates the timing of molting and metamorphosis in holometabolous insect larvae and so regulates body size by controlling the duration of growth^1,10–13^. In the third and final instar of *Drosophila* ecdysone is synthesized in a series of pulses, each of which coordinates specific developmental transitions that result in metamorphosis. The timing of these pulses is regulated by a number of environmental stimuli, including nutrition^14–17^. Nutrition acts through the IIS pathway to initiate the ecdysteroidgenesis^11^, which ultimately leads to the cessation of feeding and body growth when the pulses of ecdysone rise above a specific threshold, fixing final body size^10,12^.

At the same time, however, basal levels of ecdysone between these pulses also regulate growth rate by antagonizing systemic insulin-signaling^1,18–20^. Specifically, ecdysone is thought to act via the fat body to suppress the release of insulin-like peptides (dILPs in *Drosophila*) from insulin-producing cells (IPCs) in the brain, thereby reducing systemic IIS activity and slowing growth^19^.

The antagonistic effect of ecdysone on systemic IIS activity and growth were initially thought to be part of the developmental program that stops body growth as a larva prepares for metamorphosis^10,12,19,21^. However, more recent evidence suggests that ecdysone also plays a role in regulating nutrition-dependent growth earlier in development, independent of dILP release. This is based on the observation that low nutrition during the third larval-instar (L3) elevates basal levels of ecdysone, prior to the pulses of ecdysone that coordinate the developmental pathway to metamorphosis. This elevation in basal levels of ecdysone in turn stimulates the release of Imaginal morphogenesis protein-Late 2 (Imp-L2) from the fat body^22^. Imp-L2 is an insulin-like growth factor-binding protein that antagonizes IIS systemically and slows growth^23^, and both ecdysone and Imp-L2 are necessary for the normal effect of low nutrition on body size^22^.

An earlier study on the effect of hypoxia on ecdysone synthesis revealed that, like low nutrition, hypoxia not only affects the timing of the ecdysone pulses that initiate metamorphosis, but also elevates basal levels of ecdysone early in the third larval instar^13^. A compelling hypothesis, therefore, is that this increase in basal levels of circulating ecdysone suppress growth and final body size in low oxygen conditions. Nevertheless, a subsequent study found that hypoxia reduced expression of the ecdysone response gene *E75B*, which suggests a reduced ecdysone-induced growth restriction^6^. Thus the role of ecdysone in oxygen-regulated growth is equivocal.

Here we explicitly test the hypothesis that ecdysone regulates growth in response to hypoxia in *Drosophila,* and that it does so via mechanisms that overlap with the nutritional regulation of growth by ecdysone. We confirm that hypoxia suppresses IIS activity and has the same effect on body and organ size as low nutrition. We then show that the hypoxic suppression of both growth and IIS activity is ecdysone dependent and requires Imp-L2, but is independent of HIF-1ɑ signaling. The observation that both hypoxia and nutrition act via overlapping developmental mechanisms suggest that the effect of hypoxia on growth and body size may be a generalized systemic response to environmental stress.

## Results

### Hypoxia parallels the systemic effects of low nutrition on growth and final body size

**Figure 1:**
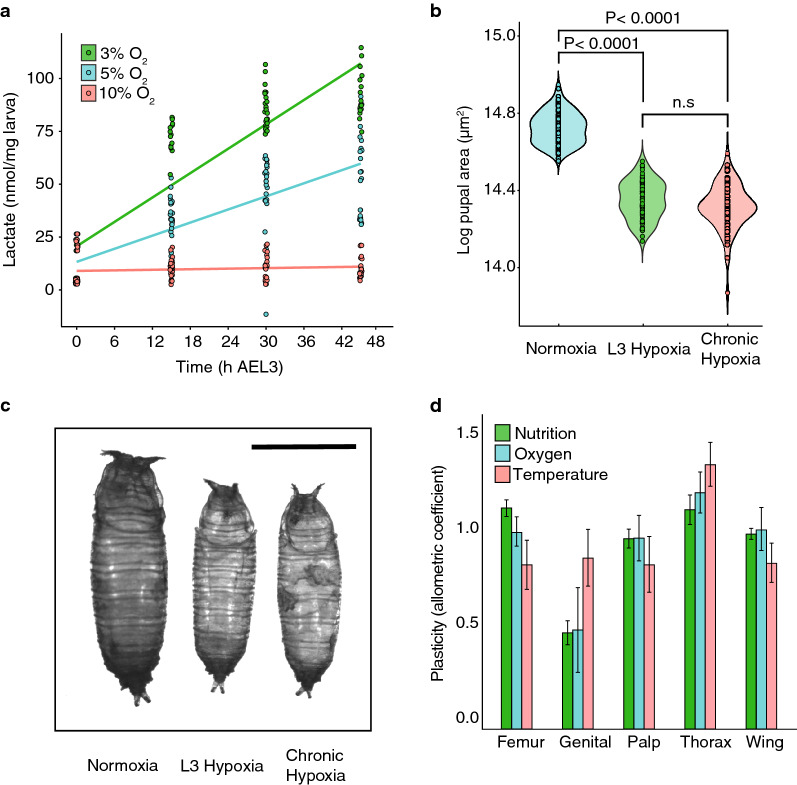
Moderate hypoxia suppresses growth without limiting aerobic respiration and in a way that parallels the effects of low nutrition on growth. (**a**) Lactate accumulation in third instar larvae transferred to different oxygen levels. Transfer to 3% and 5% O_2_ resulted in significant increase in lactate (OLS regression, P < 0.0001 for both). Transfer to 10% O_2_ had no effect on lactate levels (OL regression, P = 0.1). (**b**) Final body size (pupal case area) is reduced relative to normoxia when flies are reared in hypoxia throughout development (chronic hypoxia) or when reared in hypoxia only during the third larval instar (L3 hypoxia). Body size between treatments was compared using pooled t-tests. (**c**) Representative pictures of pupal cases of flies reared in normoxia, L3 hypoxia and chronic hypoxia. Black bar is 1 mm. (**d**) Relative plasticity of different traits to environmental variation in developmental nutrition, oxygen and temperature. The allometric coefficient captures the extent to which trait size varies relative to the body as a whole; that is, relative plasticity. An allometric coefficient of 1 indicates that the trait scales isometrically with overall body size. Error bars are 95% confidence intervals.

A simple explanation of the effect of hypoxia on growth is that it slows cell proliferation through the effects of oxygen limitation on a cell’s ability to generate ATP, as the cell switches from aerobic to anaerobic respiration. To determine what level of hypoxia induces anaerobic metabolism, we looked at the accumulation of lactate, the primary anaerobic end-product in *Drosophila*^24^, in *Drosophila* larvae transferred from 21% oxygen to 3%, 5% and 10% oxygen at ecdysis to the third larval instar (L3). We found that, while transfer to 5% and 3% oxygen resulted in strong and prolonged accumulation of lactate, no such accumulation was observed in larvae transferred to 10% (Fig. 1a). Nevertheless, larvae transferred to 10% oxygen show a reduction in overall body size of 31% (Fig. 1b,c, using pupal size as a proxy for body size), suggesting that growth suppression in 10% oxygen occurs without limits on aerobic ATP production.

Intriguingly, hypoxia during L3 alone is sufficient to reduce body size to the same size as seen in larvae exposed to hypoxia throughout development (Fig. 1b,c). This suggests that the mechanisms through which hypoxia affect final body size act primarily during the third larval instar. This parallels the effect of low nutrition on final body size, where it is slow growth during L3 that reduces final body size when nutrition is limiting^25^. Hypoxia also parallels the effects of low nutrition on body proportion. Different morphological traits in the fly have different sensitivities to changes in developmental nutrition^26^. Wing-, mouthpart- and leg-size all show moderate sensitivity to changes in nutrition while genital-size shows low sensitivity (Fig. 1d). This is in contrast to the effect of temperature on trait size, where wing-size is highly sensitive to changes in temperature, but the leg-size is relatively insensitive (Fig. 1d). We found that the pattern of trait sensitivity to changes in oxygen are the same as for changes in nutrition, but very different than for changes in temperature (Fig. 1d).

Collectively, these data suggest that the effect of 10% oxygen on growth and final body size is primarily mediated through a systemic mechanism rather than due to the effects of low oxygen on cellular respiration, and that this mechanism overlaps with the physiological mechanisms that slow growth and reduce final body size in response to low nutrition. Because we observed the same effects on final body size in larvae exposed to 10% O_2_ during the third larvae instar as larvae exposed to hypoxia throughout development, unless otherwise stated, all our subsequent experiments were on larvae transferred from 21 to 10% oxygen at the beginning of the third larval instar.

### The IIS-pathway is suppressed under hypoxic conditions

**Figure 2:**
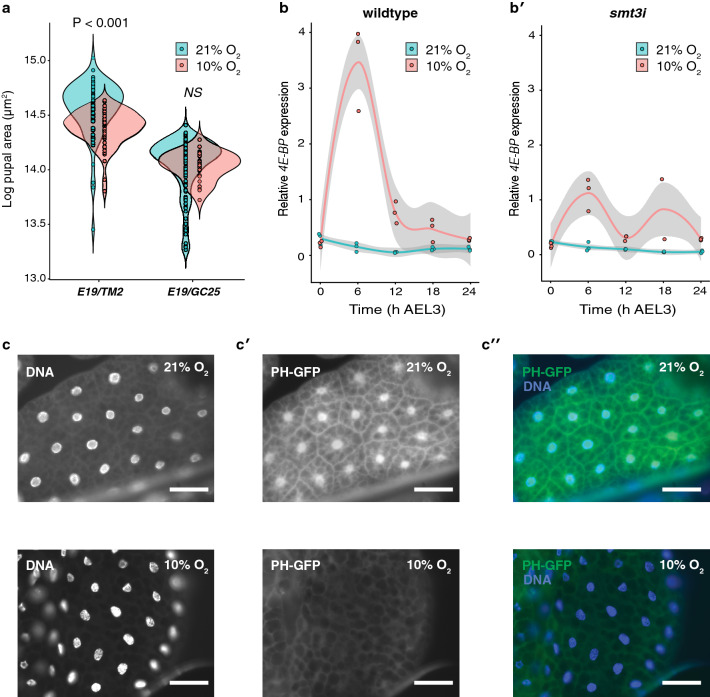
Moderate hypoxia reduces body size by suppressing IIS activity. (**a**) Suppression of the Insulin-Signaling pathway is necessary to inhibit growth under hypoxia. Adult body size shown in log pupal case area in flies functional IIS (E19/TM2) and flies with suppressed IIS (E19/GC25). Mean pupal size for each genotype across oxygen levels was compared using a pooled t-test. NS: not significant at P > 0.05. (**b**) Hypoxia increases expression of 4E-BP in the first 24 h after ecdysis to the third larval instar (AEL3) relative to normoxic controls. (**b’**) This increase in expression is alleviated in smt3i larvae, which have reduced ecdysone levels. (**c,c’,c’’**) TGPH membrane localization in the fat body cells under hypoxic and normoxic conditions. TGPH localizes to the cell membrane when IIS-pathway activity is high. White bars represent 100 µm. Grey bands are 95% confidence limits around the mean.

The IIS pathway is the primary regulator of growth in response to low nutrition, whereby under low nutrition reduces level of circulating dILPs and suppressing the activity of the pathway in proliferating cells. An earlier study demonstrated that 5% O_2_ suppresses systemic IIS activity^6^, but larvae are respiring at least partially anaerobically at this oxygen level (Fig. 1a). We therefore explored whether IIS activity is suppressed in moderately hypoxic conditions (10% O_2_), sufficient to reduce final body size but where aerobic respiration is maintained. We first looked at the effect of 10% O_2_ on flies carrying a mutation of *InR* that causes constitutive suppression of IIS activity (*InR*^*E19/GC25*^). Hypoxia had no effect on final body size in these flies (Fig. 2a), supporting the hypothesis that changes in IIS activity are necessary for the growth response to moderate hypoxia. We then measured the mRNA levels of *4E-BP* under normoxic (21% O_2_) and hypoxic (10% O_2_) conditions (Fig. 2b). *4E-BP* is a target the IIS pathway and its expression is high when IIS activity is suppressed^19^. Consistent with previous studies on the effects of severe hypoxia (5% O_2_) on IIS^6^, we observed an increased *4E-BP* expression when larvae were reared in moderate hypoxic conditions. Finally, we looked at the effect of hypoxia on Pi3K activity using the TGPH reporter construct, which localizes to the cell membrane when Pi3K and IIS-activity is high^27^. We found that Pi3K activity was low at 10% O_2_ compared to 21% O_2_ (Fig. 2c,c’,c’’). Collectively, these data show that the IIS-pathway is suppressed under moderately hypoxic conditions that do not induce anaerobic respiration.

### Ecdysone is required to suppress growth and IIS activity under moderate hypoxia

Previous studies indicate that levels of circulating ecdysone are elevated in third larval instar under moderate hypoxia^13^. Further, elevated levels of ecdysone are known to reduce body growth^19,22^. We therefore tested the hypothesis that hypoxic growth-reduction is mediated through elevated levels of ecdysone. We first generated larvae in which the prothoracic gland was genetically ablated, referred to as PGX larvae (Supp. Fig. 1). This was achieved by combining the temperature sensitive repressor of GAL4, GAL80ts, with a prothoracic gland-specific GAL4 (*phm-GAL4*) to drive an apoptosis-inducing gene (*UAS-GRIM*). GAL80^ts^ is active at 17 °C, where it represses GAL4 action, but inactive above 25 °C, which allows *phm-GAL4* to drive expression of *UAS-GRIM* and ablate the PG^28^. We then compared growth of PGX and control larvae (which were genetically identical but lacked *UAS-GRIM*) in normoxic and hypoxic conditions. We found a significant interaction between the effects of oxygen level and genotype (PGX v. control) on growth, such that loss of the PG increased growth rate in hypoxic conditions (Supp. Fig. 1). This demonstrates that growth suppression by hypoxia requires a functional PG in *Drosophila*.

**Figure 3:**
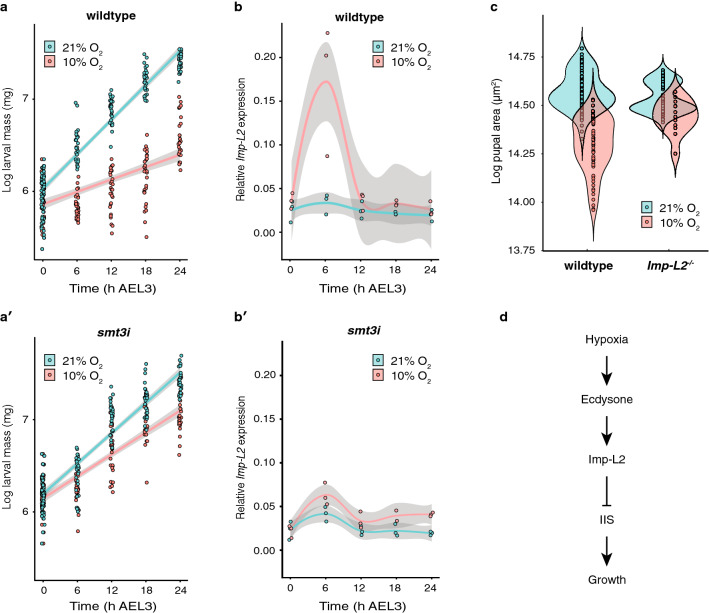
Suppression of ecdysone synthesis alleviates hypoxic growth reduction via *Imp-L2*. (**a**) Growth rate of wildtype third instar larvae is substantially reduced in hypoxia relative to normoxic controls. (**a’**) The effect of hypoxia on growth rate is significantly reduced in *smt3i* flies (GLM, *P*_time*genotype*oxygen_ < 0.0001). (**b**) Expression of *Imp-L2* is elevated 6 h AEL3 in wildtype hypoxic larvae. (**b’**) Expression of *Imp-L2* is reduced in *smt3i* hypoxic larvae. (**c**) Loss of Imp-L2 (*Imp-L*^*−/−*^), almost eliminates the effect of hypoxia on final body size (*t*-test, *P*_genotype*oxygen_ < 0.0001). (**d**) Model of the physiological mechanism by which hypoxia regulates growth and final body size in *Drosophila*. Grey bands are 95% confidence limits around the mean.

To confirm our hypothesis that ecdysone synthesis itself is required to suppress growth under hypoxic conditions, we adopted a more targeted approach to disrupt ecdysone synthesis without genetic ablation of the PG. We achieved this by silencing the *Drosophila* Small Ubiquitin Modifier (SUMO) 2/3 homologue (smt3) in the PG through RNAi knockdown (*phm>smt3.RNAi*, referred to as *smt3i*), which prevents the production of high levels of ecdysone in the third larval instar (Supp. Fig. 2)^29,30^. We again compared growth of these *smt3i* larvae with control larvae in normoxic and hypoxic condition. As for the PGX experiment, we found a significant interaction between the effects of oxygen level and genotype (*smt3i* v. control) on growth, such that suppression of ecdysone synthesis increased growth rate in hypoxic conditions (Fig. 3a,a’). These data confirm that ecdysone synthesis is necessary for complete growth suppression in moderate hypoxia.

We also tested whether inhibiting the elevation of ecdysone under hypoxic conditions also inhibits the suppressive effects of hypoxia on IIS activity, using expression of *4E-BP* as an indicator of IIS activity (Fig. 2b’). We observed that in *smt3i* larvae with suppressed ecdysone synthesis, hypoxia no longer increased the expression of *4E-BP*. The effect of hypoxia-elevated ecdysone on IIS activity appears to be independent of HIF-signaling, however. To assay HIF-signaling activity we measured the expression HIF-1α Prolyl Hydroxylase (Hph), a transcriptional target of HIF-1α^4^. While *Hph* expression was elevated under hypoxia, this elevation was maintained when ecdysone synthesis was suppressed in *smt3i* larvae (Supp. Fig. 3). Collectively these data indicate that ecdysone synthesis is necessary to suppress activity of the IIS-pathway and body growth under moderate hypoxia, and does not appear to do so via HIF-1α signaling.

### Imp-L2 is required to reduce final body size under hypoxia

We next asked how ecdysone suppresses IIS and growth under hypoxic conditions. The inhibitory effects of elevated ecdysone on growth at low nutrition are mediated by Imp-L2, an insulin binding protein that inhibits systemic IIS. We therefore tested the hypothesis that Imp-L2 mediates the effect of ecdysone on growth and final body size in hypoxia. First, we assayed the expression of *Imp-L2* throughout early L3 and found that it was transiently elevated under hypoxic conditions but not normoxic conditions (Fig. 3b). We then explored whether this hypoxia-induced increase in *Imp-L2* expression is ecdysone dependent by looking at *Imp-*L2 expression in *smt3i* larvae reared in hypoxia and normoxia. We found that suppression of ecdysone synthesis suppressed the expression of *Imp-L2* early in L3 (Fig. 3b’). To confirm that elevation in *Imp-L2* expression in hypoxia is responsible for the effect of hypoxia on body size, we reared *Imp-L2* null mutant fly line (*Imp-L2*^*Def*42^) in hypoxic and normoxic conditions and looked at the effect on final body size. We found that loss of *Imp-L2* increased final body size in hypoxia, but had no effect on final body size in normoxia, relative to controls (Fig. 3c). Thus Imp-L2 is necessary for growth suppression in hypoxic conditions.

## Discussion

Collectively our data support a link between ecdysone, growth and final body size at low oxygen levels, where moderate hypoxia increases basal ecdysone synthesis, which up-regulates expression of *Imp-L2*, which in turn suppresses growth and reduces final body and organ sizes by suppressing IIS (Fig. 3d). The mechanisms that regulate growth and final body size in response to low oxygen therefore overlap with those that regulate growth and final body size in response to low nutrition, and explains why both environmental factors have the same effect on the size of individuals traits (Fig. 1d).

Previous studies have implicated the fat body as an oxygen sensor that regulates growth and survival in response to low oxygen in *Drosophila*^6,31^. Two signaling pathways appear to be involved: HIF-1α signaling and TOR signaling.

HIF-1α is a transcription factor that is activated by hypoxia through its oxygen-dependent degradation by HIF-1α Prolyl Hydroxylase (Hph)^4,5,32–34^. Inducing hypoxia in the fat body alone, by limiting tracheal growth, represses dILP2 secretion from the brain, lowers systemic IIS and reduces growth, and this effect is phenocopied in normoxia by activating HIF-1α in the fat body^6^. Importantly, knock-down of HIF-1α in the fat body at low oxygen levels alleviates the effects of hypoxia on dILP2 secretion and ostensibly on growth^6^.

TOR-signaling regulates cell growth and proliferation in response to both cellular levels of amino acids and through crosstalk with IIS. Low oxygen suppresses TOR-signaling in the fat body via Hph, and this is necessary for hypoxia tolerance and survival to adulthood^6,31^. In normoxia, suppression of TOR-signaling in the fat body reduces the release of dILP2 from the brain and slows growth^35^, which suggests that the suppression of TOR-signaling by hypoxia should have the same effect. Activating TOR-signaling in the fat body does not, however, relieve the suppressive effects of hypoxia on growth^6^, so while suppression of TOR signaling may be sufficient to slow growth in hypoxia, it does not appear to be necessary.

The effect of hypoxia and HIF-signaling in the fat body on growth is thought to be independent of ecdysone, since neither enhances the expression of *E75B*, an ecdysone response gene^6^. Our results implicating ecdysone in the oxic regulation of growth but not via HIF-1α signaling therefore appears inconsistent with these previous studies. There are number of possible explanations for these inconsistencies:

First, hypoxia may affect growth, IIS activity and final body size through two mechanisms: one via ecdysone synthesis by the PG regulating Imp-L2 and a second via HIF-signaling in the fat body regulating dILP2. This parallels the effect of low nutrition on larval growth, which also appears to function both through the effects of elevated ecdysone and Imp-L2, and through suppression of diLP2 release through reduced TOR-signaling in the fat body^31^. Support for this explanation comes from the observation that suppression of ecdysone synthesis does not completely alleviate the effects of low oxygen on growth rate, so that hypoxic PGX and *smt3i* larvae do not grow at the same rate as larvae reared in normoxia. Thus some mechanism other than elevated ecdysone appears to be suppressing growth in these larvae.

Second, the differences in ours and previous results may reflect differences in the levels of hypoxia larvae are exposed to. It is possible that in moderate hypoxia (10% O_2_), when larvae are still able to respire aerobically, growth is suppressed by elevating ecdysone levels, while at more severe hypoxia (5% O_2_), growth is (further) suppressed by HIF-signaling in the fat body. This is further supported by the observation that TOR signaling is only suppressed at oxygen levels between 2 and 6%^31^. The observation that whole-body *Hph* expression levels are elevated at 10% O_2_, however, indicates that there is nevertheless some HIF-signaling at this level of hypoxia (Supp Fig. 3).

Finally, the observation that hypoxia reduces *E75B* expression—which argues against the involvement of ecdysone in the hypoxic regulation of growth—may reflect changes in the dynamics of ecdysone synthesis at low oxygen rather than its suppression. During the third larval instar, timed pulses of ecdysone –interspersed with lower basal levels of ecdysone—drive key developmental events that lead to metamorphosis^12,36^. The initiation of these pulses coincides with attainment of a critical weight^37^, at which point ecdysone synthesis becomes self-regulated by positive and negative feedback loops^38^. Previous studies have shown that low nutrition increases basal levels of ecdysone to slow growth but delays attainment of critical weight and hence retard the pulses of ecydysone that end in metamorphosis^22,25,39^. The same appears to be happening in hypoxia: we measured the expression of *E74B*, an ecdysone-response gene that increases in expression in response to the ecdysone pulse that drives salivary gland histolysis, ~ 20 h after ecdysis to the third instar. We found that low oxygen suppressed the expression of *E74B* in the first 24 h of the third larval instar (Supp. Fig. 2a), consistent with the observation that hypoxia delays attainment of critical weight and the timing of the ecdysone pulses that result in metamorphosis^13^. This would also explain why hypoxia appears to reduce *E75B* expression. *E75B* is normally expressed in response to the ecdysone pulse that drives pupariation, ~ 32 h after ecdysis to the third instar. The reduced expression of *E75B* at a single time point in hypoxic larvae may therefore reflect a delay in this pulse rather than a general suppression of ecdysone synthesis. Nevertheless, the delay in the pulses of ecdysone cannot account for slow growth in hypoxia since loss of ecdysone synthesis increases growth rate at low oxygen. Thus, it appears to be the elevated basal levels of ecdysone that slow growth at low oxygen levels^13^.

Whether the elevated levels of ecdysone observed under hypoxia and low nutrition—and that are necessary for growth suppression—are due to increase in ecdysone synthesis versus degradation is unclear. One route to metabolism of ecdysteroids in insects is through oxidation by ecdysone oxidase. This reaction requires oxygen, and so a simple hypothesis is that, in hypoxic conditions, ecdysone oxidase is unable to metabolize ecdysone, increasing basal ecdysone levels. We explored whether expression of ecdysone oxidase was elevated under hypoxic conditions, and found that it was not (Supp. Fig. 4). However, since this hypothesis proposes that ecdysone oxidase activity is regulated by oxygen levels, we may not necessarily expect to also see a change in *ecdysone oxidase* expression. Alternatively, the increase in circulating ecdysone in hypoxia may be a consequence of an increase in ecdysone synthesis. The observation that hypoxia delays the pulses of ecdysone that precede pupariation indicates that low oxygen impacts ecdysteroidgenesis. Whether hypoxia can simultaneously increase basal synthesis of ecdysone while delaying the initiation of the pulses of ecdysone that drive metamorphosis is an open question. Further elucidation of the effect of oxygen on the expression and activity of genes involved in ecdysone synthesis and metabolism are clearly needed. One additional complexity is that, while our data place IIS downstream of ecdysone in the regulation of growth in response to hypoxia, IIS is also a positive regulator of ecdysteroidgenesis^11^, with suppression of IIS in the PG suppressing basal levels of ecdysone^19^. What role this negative feedback loop plays in the regulation of growth and developmental timing in response to both low nutrition and hypoxia requires further examination.

Both hypoxia and low nutrition slow growth rate via ecdysone signaling, both suppress IIS via Imp-L2, and both affect body proportion in the same way. However, the two environmental factors do not have identical effects on growth and development in *Drosophila*. Final body size in *Drosophila* is in part regulated by critical weight^40,^ which is reduced in low oxygen^13^ but not low nutrition^25^. Final body size is also dependent on the duration of the terminal growth period (TGP) between attainment of critical weight and pupariation, which is reduced in starved larvae^41^ but increased in hypoxic larvae^13^ (although larvae on low nutrition may also increase their TGP^42^). Nevertheless, the primary mechanism by which adult body size is suppressed in both hypoxic and low nutrition conditions is by a reduction in growth rate during the TGP, which appears to be regulated by the same mechanisms for both environmental conditions.

In conclusion, our data support a model where hypoxia suppresses IIS and growth in *Drosophila* via the hormone ecdysone and the insulin-binding protein Imp-L2 (Fig. 3d). The suppressive effect of hypoxia on growth, however, is highly evolutionarily conserved and observed in myriad animals. Interestingly, hypoxia due to acute respiratory distress in children leads to an increase in serum growth hormone concentrations, a decrease in serum IGF-1, and an increase in serum IGF Binding Protein-1^43^. An open question, therefore, is whether other animals also utilize systemic mechanisms in general, and steroid and insulin-signaling specifically to slow growth under conditions of low oxygen. Further, our data indicate that both low oxygen and low nutrition affect growth using overlapping mechanisms. Animals such as *Drosophila* grow and develop facing a multitude of physiological stresses and environmental perturbations throughout development. The observation that the insulin- and ecdysone-signaling pathways are important in response to at least two environmental stresses, suggests that these two pathways might be part of a more general response to environmental stress in *Drosophila*, regardless of the nature of the stress. Whether other animals also utilize a general growth regulatory mechanism to respond to general environmental stress requires further study.

## Materials and methods

### *Drosophila* stocks

Flies were reared on standard *Drosophila* yeast cornmeal molasses medium. The following fly strains were used for this study: the isogenic *RNAi* control (*60,000*) was obtained from the Vienna *Drosophila* RNAi Center, *phm-GAL4* (a gift from Michael O’Connor), *UAS-smt3i*^20^ and *tub-Gal80*^*ts*^*;phm-Gal4* (gifts from Rosa barrio), *UAS-Grim* (a gift from Christen Mirth), *Imp-L2*^*def42*^ (a gift from Seogang Hyun), *P{tGPH}* (BDSC 8164), *InR*^*E19*^ (BDSC 9646), *InR*^*GC25*^ (BDSC 9554), and *w*^*1118*^ (BDSC 6326).

### Hypoxia treatment

Unless otherwise stated, all flies were reared on standard cornmeal-molasses medium at 25 °C, 21% atmospheric O_2_ level until ecdysis to the third instar. Larvae were then either maintained at 21% O_2_ (normoxic flies) or moved to 10% O_2_ (hypoxic flies).

### Lactate assay

The lactate assay was conducted by measuring NADH produced from lactate in presence of lactate dehydrogenase (LDH) and NAD^+^^44^ NADH was measured using a Farrand Optical Components & Instruments Standard Curve System Filter Fluorometer (Valhalla, NY, USA) set at 360 nm excitation wavelength and 460 nm emission wavelength.

#### Buffer preparation

To make up 100 ml of 2 × Hydrazine-Tris buffer, 20 ml of a 1.0 M stock solution of Tris-Base was added to 20 ml of ddH_2_O. 10 ml of 20 M liquid hydrazine and 2.25 ml of a 125 mM stock solution of EDTA were then added to the solution, which was then bought to a pH of 10.0. ddH_2_O was added to a final volume of 100.0 ml. Finally, 5 mM of NAD^+^ was added to the buffer solution right before the assay.

#### Sample preparation

50 µL of HClO_4_ (17.5%) per mg of larva was added to each Eppendorf tube containing larval sample to stop metabolism and ground with a pestle. The protein was centrifuged down (1 min at 14,000*g*). To neutralize the acidified extract, 1.0 ml of the supernatant was added to 0.225 ml KOH + MOPS solution ([KOH] = 2.0 N and [MOPS] = 0.3 M).

#### Assay procedure

Each cuvette contained 20 µL of sample, 100 µL of Hydrazine-Tris buffer with 5 mM NAD^+^, and 3% LDH in 90 µL ddH_2_O. Cuvettes were incubated at room temperature for 30 min, and then read in the fluorometer. The amount of lactate in the sample was calculated from the linear relationship between the NADH produced and lactate levels in known standard solutions.

### Nutritional, thermal and oxic plasticity

We measured the effect of developmental nutrition, temperature and oxygen level on the size of five traits in wild type (*OreR*) male *Drosophila*: wing area, leg length, maxillary palp area, and the area of the posterior lobe of the genital arch. Data for the effect of nutrition and temperature on body size was taken from a previously published paper^26^. To measure the effect of oxygen level on trait size, flies were reared throughout development at 5%, 10% or 21% O_2_. To determine the multivariate allometric coefficient under each condition, we calculated the loadings of the first eigenvector of the variance–covariance matrix for trait size, as trait size varied in response to each environmental factor. Multiplying the loadings by √*n* (where *n* is the number of traits) gives the allometric coefficient for each trait against a measure of overall body size, and is a measure of relative trait plasticity.

### Constitutive suppression of IIS

*InR*^*E19*^*/InR*^*GC25*^ have a temperature-sensitive suppression of InR activity, such that at 17 °C, InR activity is normal and at 24 °C InR activity is suppressed^25^. *InR*^*E19*^*/InR*^*GC25*^ (experimental) and *InR*^*E19*^*/TM2* (control) larvae were reared at 17 °C under standard conditions until ecdysis to the third instar, and where then moved to 10% O_2_ at 24 °C and left to pupate. We collected digital images of the pupal cases and measured the area of the pupal case when viewed from the dorsal aspect using ImageJ^45^.

### TGPH localization

*P{tGPH}* larvae were reared as described above until ecdysis to the third larval instar and either maintained at 21% O_2_ or transferred to 10% O_2_. After 12 h, larvae were collected, and their fat bodies were dissected, fixed, stained and imaged using previously published protocols^25^.

### Measurements of growth in PGX and *Phm* > *Smt3i* larvae

For the PGX experiment, we crossed a *tub-GAL80*^*ts*^*, phm-GAL4* strain with *UAS-Grim* to generate PGX progeny (*tub-GAL80*^*ts*^*, phm-GAL4, UAS-GRIM*)^30^. GAL80^ts^ is a repressor of GAL4 active at temperatures lower than 22 °C, but is not functional above 25˚C, allowing *phm-GAL4* to drive expression of *UAS-Grim* and promoting cell death in the prothoracic gland alone. Females were left to oviposit on 50 mm diameter Petri dishes contain standard cornmeal-molasses medium for 24 h at 17 °C at a density of ~ 100 eggs per plate. PGX Larvae were left to develop at 17 °C, 21% O_2_ and staged at the third larval instar by collecting newly ecdysed third instar larvae and transferring them to fresh food plates, in 3 h cohorts. Larvae were then transferred to 29 °C at either 21% O_2_ or 10% O_2_, disabling the suppressive activity of GAL80^ts^ and ablating the PG^28,46^. Larval mass was measured at ecdysis to the third larval instar and 18 h later, by removing larvae from the food plates and massing them individually on a Mettler Toledo XPR36 Micro-Analytical Balance. Control larvae were the progeny of *tub-GAL80*^*ts*^*, phm-GAL4* and *w*^*1118*^ and were treated identically.

For the *Smt3i* experiment, we crossed *UAS-smt3i*^29^ with *phm-GAL4* flies to generate progeny in which sumolation in the PG is suppressed, inhibiting ecdysone synthesis in the third larval instar^29,30^. Females were allowed to oviposit as described above and larvae were reared at 25 °C and 21% O_2_. Larvae were then staged at the ecdysis to L3 into 3 h cohorts and either transferred to 10% O_2_ or maintained at 21% O_2._ We massed larvae every 6 h up to 24 h after ecdysis to the third larval instar (AEL3) by removing thirty larvae from the food plates and massing them individually on a Mettler Toledo XPR6UD5 UltraMicrobalance. Larvae were not returned to the food plate but were used to measure gene expression (see below). Control larvae were the progeny of *phm-GAL4i* and *60,000* and were treated identically.

### Quantitative PCR (qPCR)

Larvae were collected every 6 h for the first 24 h of the third larval instar, as described above, and preserved in groups of ten in RNAlater (ThermoFischer Scientific). RNA was extracted using Trizol (Invitrogen) and treated with DNase I (ThermoFischer Scientific). RNA was quantified with a NanoDrop One (ThermoFischer Scientific) and reverse transcribed with High-Capacity cDNA Reverse Transcription Kit (Applied Biosystems). Quantitative RT–PCR was conducted using PowerUp SYBR Green Master Mix (Applied Biosystems) and measured on an QuantStudio 3 Real-Time PCR system (Applied Biosystems). mRNA abundance was calculated on three biological replicates of ten larvae, using a standard curve and normalized against expression of ribosomal protein 49 (RP49). Standard curves were generated using seven serial dilutions of total RNA extracted from the isogenic control line (60,000): 5 × 1st instar larvae, 5 × 2nd instar larvae, 3rd instar larvae (male), 5 × pupae (male), 5 × adult flies (male). Primer sequences used in the study were (5’- 3’):

- RP49 Forward: AAG AAG CGC ACC AAG CAC TTC ATC
- RP49 Reverse: TCT GTT GTC GAT ACC CTT GGG CTT
- Imp-L2 Forward: GCG CGT CCG ATC GTC GCA TA
- Imp-L2 Reverse: TTC GCG GTT TCT GGG CAC CC
- 4EBP Forward: CAG ATG CCC GAG GTG TAC T
- 4EBP Reverse: GAA AGC CCG CTC GTA GAT AA
- E74B Forward: ATC GGC GGC CTA CAA GAA G
- E74B Reverse: TCG ATT GCT TGA CAA TAG GAA TTT C
- Hph Forward: GGC AAC CAA AAA GTG AAA TCC A
- Hph Reverse: TGA CCG AAG TTG TAG TAG CCG
- EO Forward: CCG ATT CCG ATG ACT GG
- EO Reverse: CGC TGG CAA TTC CGG CAT AA.

## Supplementary Information

Supplementary Data.

Supplementary Figures.

## Data Availability

All data that support all the experimental findings in this article is available in the Supplementary Data File provided.
