# Supplementary material for: The steroid hormone ecdysone regulates growth rate in response to oxygen availability
Source: Sci Rep. 2022 Mar 18;12:4730. doi: 10.1038/s41598-022-08563-9 (PMC8933497; doi:10.1038/s41598-022-08563-9)
Supplement: Supplementary file 2 — Supplementary Figures. [file 41598_2022_8563_MOESM2_ESM.pdf]

Title: The steroid hormone ecdysone regulates growth rate in response to oxygen availability

Authors: George P. Kapali, Viviane Callier, Samuel J.L. Gascoigne, Jon F Harrison, Alexander W. Shingleton

Supplementary Figures:

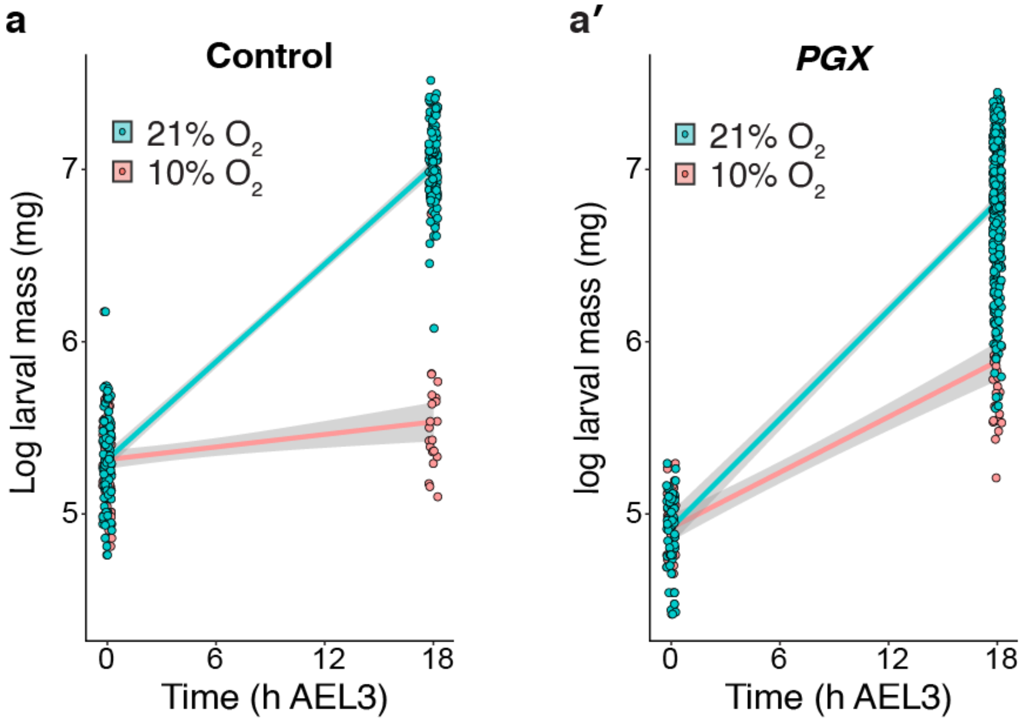

Supplementary Figure 1: Ablation of the PG (site of ecdysone production) alleviates negative effects of hypoxia on growth. (**a**) 3rd instar larval weight in log-mass over 18 hours in wildtype flies and (**a'**) *PGX = phm>grim;tubGAL80*^{TS} flies (GLM, $P_{time*genotype*oxygen}$ <0.0001).

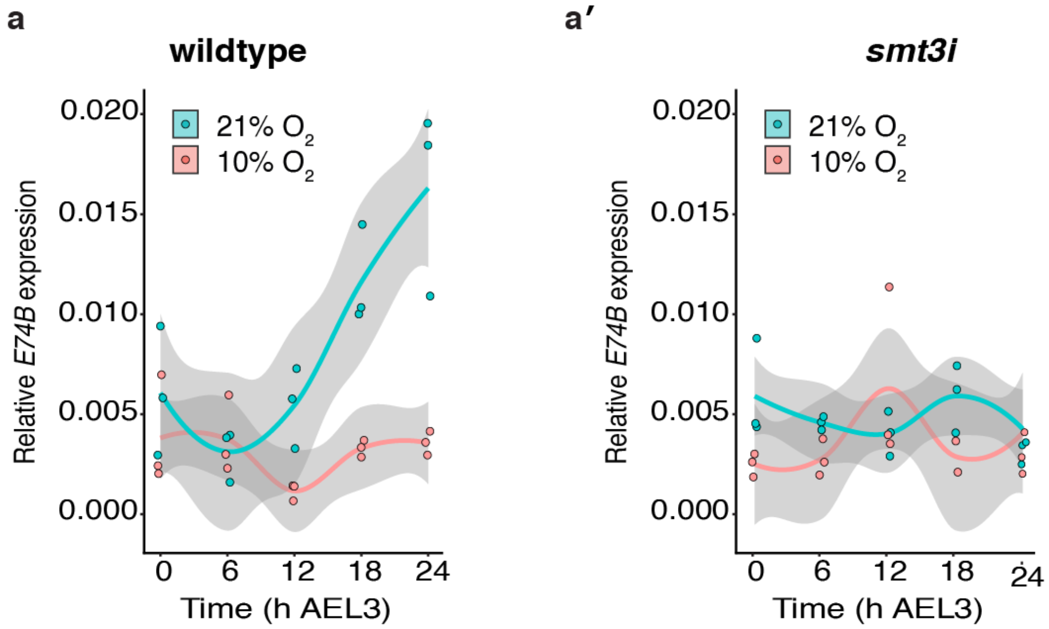

Supplementary Figure 2: *E74B* mRNA expression. (**a**) Transcript levels of *E74B* over 24 hours after L3 eclosion in control larvae were determined with qPCR, normalized to RP49 mRNA. (**a'**) Transcript levels of *E74B* in *smt3i* larvae. Grey bands are 95% confidence limits around the mean.

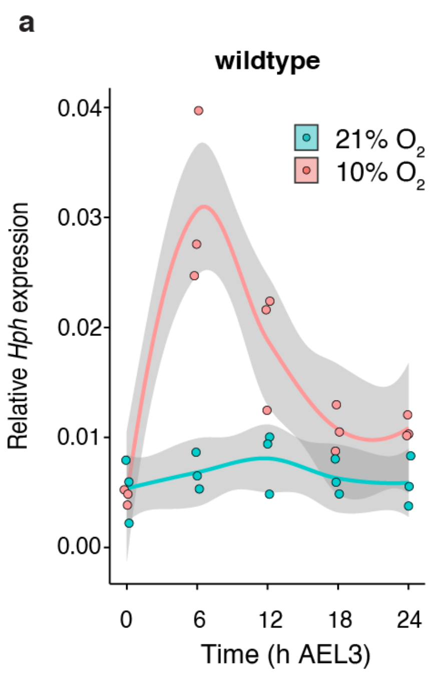

Supplementary Figure 3: *Hph* mRNA expression. (**a**) Transcript levels of *Hph* over 24 hours after L3 eclosion in control larvae were determined with qPCR, normalized to RP49 mRNA. (**a'**) Transcript levels of *Hph* in *smt3i* larvae. Grey bands are 95% confidence limits around the mean.

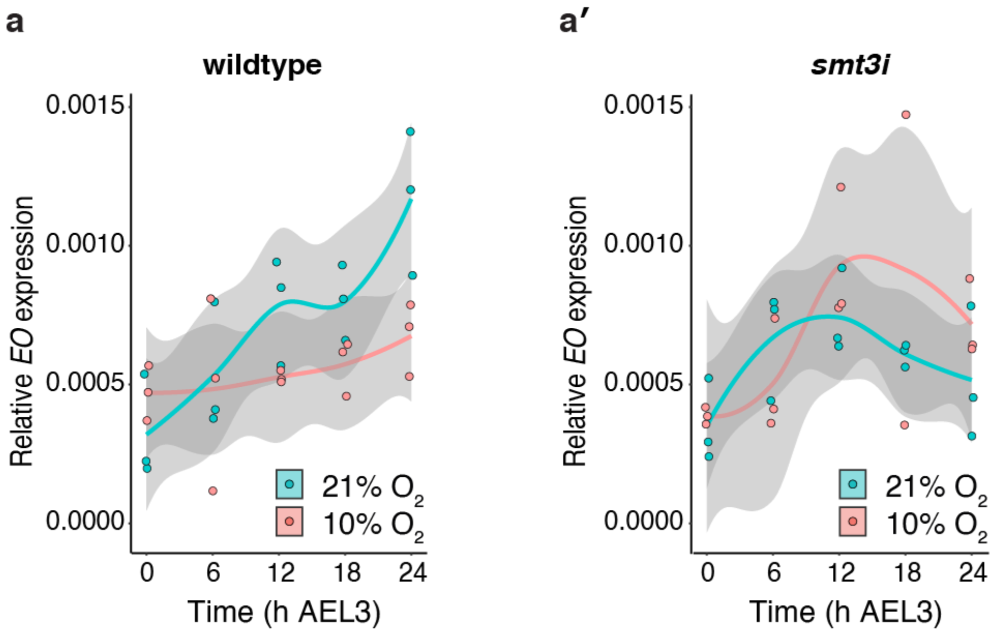

Supplementary Figure 4: *EO* mRNA expression. (**a**) Transcript levels of *EO* over 24 hours after L3 eclosion in control larvae were determined with qPCR, normalized to RP49 mRNA. (**a’**) Transcript levels of *EO* in *smt3i* larvae. Grey bands are 95% confidence limits around the mean.
